# Characteristics of childhood-onset systemic lupus erythematosus in pregnancy and its association with pregnancy outcomes: a retrospective comparative cohort study

**DOI:** 10.1186/s12958-022-00954-x

**Published:** 2022-05-19

**Authors:** Zhi-Ju Li, Hao-Yue Hu, Zi-Ling Ding, Zi-Wei Bian, Ying-Hua Xu, Hui-Ting Wen, Ya-Li Qu, Jin-Dong Wang, Xiao-Li Huang, Dong Li, Jing Li, Gui-Fang Hu

**Affiliations:** 1 Department of Epidemiology, School of Public Health, Southern Medical University, No.1846, North of Guangzhou Avenue, Guangzhou, 510515 Guangdong China; 2 Department of Obstetrics and Gynecology, Nanfang Hospital, Southern Medical University, No.1846, North of Guangzhou Avenue, Guangzhou, 510515 China; 3 School of Public Health, Southern Medical University, Guangzhou, Guangdong China

**Keywords:** Systemic lupus erythematosus, Pregnancy, Childhood-onset, Pregnancy outcomes

## Abstract

**Background:**

Disease situations are more aggressive in patients with childhood-onset systemic lupus erythematosus (cSLE) than in those with adult-onset SLE (aSLE). However, information on pregnant women with cSLE and its association with pregnancy outcomes is limited. This study aimed to compare pregnancies in patients with cSLE vs. aSLE, and further analyse the characteristics of cSLE in pregnant women and explore its association with adverse pregnancy outcomes.

**Methods:**

Altogether, data of 167 pregnancies from 150 women, including 22 pregnancies with cSLE and 145 pregnancies with aSLE, were retrospectively analysed. Characteristics and disease activity were compared between the cSLE and aSLE groups during pregnancy. Associations between cSLE and the risk of active SLE (SLEPDAI > 4), active lupus nephritis (LN), and adverse pregnancy outcomes were analysed using logistic regression.

**Results:**

The cSLE group had a higher incidence of active SLE (12/22 vs. 30/145, *P* = 0.001) and active LN (11/22 vs. 26/145, *P* = 0.001) than the aSLE group. In the multivariable analysis, cSLE was a risk factor for active SLE and active LN during pregnancy, with *ORs* of 4.742 (95%*CI* 1.678–13.405, *P* = 0.003) and 4.652 (95%*CI* 1.630–13.279, *P* = 0.004), respectively. No significant association between cSLE and the risk of composite adverse gestational outcomes was identified after sequentially adjusting pre-pregnancy characteristics and pregnancy factors (*P* > 0.05).

**Conclusion:**

Disease activity of women with cSLE in pregnancy was more aggressive than that of women with aSLE, which was similar to the characteristics of non-pregnant women with SLE. cSLE might have indirect effects on the risk of adverse pregnancy outcomes through LN and active disease. Therefore, closely monitoring patients with cSLE during pregnancy is crucial.

## Background

Systemic lupus erythematosus (SLE) is an autoimmune disease involving multiple systems of the body that mainly affects women of reproductive age [1, 2]. Pregnancy was considered a contraindication to SLE in the past; however, patients with SLE are more likely to have a smooth and healthy pregnancy with standardised treatment and management [3, 4]. Systematic reviews and meta-analyses have demonstrated that SLE was associated with the risk of pregnancy outcomes, such as foetal loss, preterm birth, infants with low birth weight (LBW), and hypertensive disorders in pregnancy (HDP) [5, 6]. Thus, SLE remains a severe risk factor for pregnancy. Moreover, increasing studies have shown that conditions including patients in remission for < 6 months before pregnancy, lupus nephritis (LN), new-onset SLE, disease flare, low complement, and antiphospholipid syndrome might increase the risk of adverse pregnancy outcomes in patients with SLE [7–12].

It is estimated that 10–20% of patients with SLE are diagnosed in childhood, where kidney involvement occurs in > 50% of children [13, 14]. There is a deeper understanding of the differences in disease manifestations, medication use, disease severity, and health-related quality of life between patients with childhood-onset SLE (cSLE) and those with adult-onset SLE (aSLE) [15, 16]. Systemic manifestations, severe organ involvement, especially LN, and risk of mortality are more common in patients with cSLE than in those with aSLE [17, 18]. However, information on pregnant women with cSLE and its association with pregnancy outcomes is limited. This retrospective cohort study aimed to compare the characteristics and pregnancy outcomes between the cSLE and aSLE pregnant women, and further analyse the characteristics of cSLE in pregnant patients and explore its association with adverse pregnancy outcomes.

## Methods

### Patients and study design

**Fig. 1:**
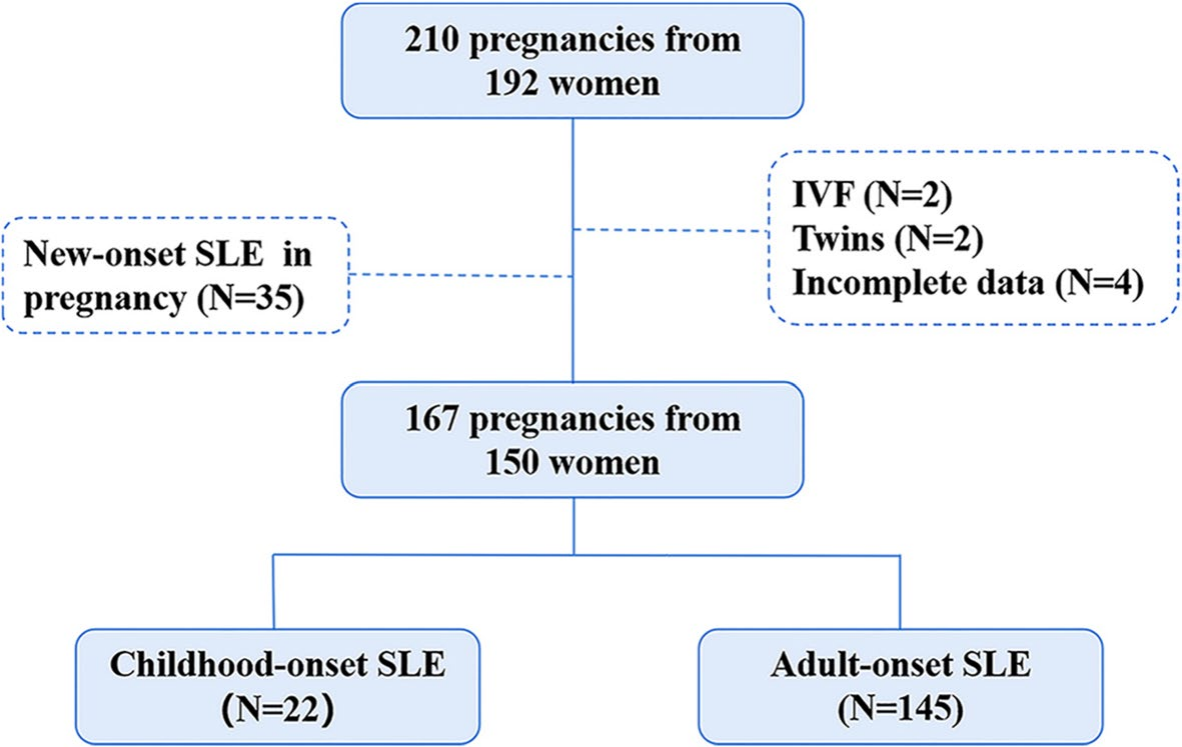
Flowchart for patient enrollment and grouping

As shown in Fig. 1, 210 pregnancies with SLE were identified according to the 1997 American College of Rheumatology (ACR) revised criteria [19] for SLE in our retrospective cohort study. From January 2010 to January 2020, they were regularly followed, evaluated, and managed by both rheumatologists and obstetricians in Nanfang Hospital, which is a comprehensive third-level grade-A hospital in South China. Of the 210 pregnancies, two IVF pregnancies, two twin pregnancies, four pregnancies with incomplete data, and 35 pregnancies with new-onset SLE during pregnancy were excluded. Finally, 167 pregnancies from 150 women were included in our study. The participants were divided into the cSLE (< 18 years old) group and the aSLE (≥18 years old) group based on the recommended age cut-off of 18 years [20]. Demographic profiles [gestational age, pre-pregnancy body mass index (BMI), native place, and employment profile], maternal history of atopy, disease history (SLE duration, a history of LN, SLE activity before pregnancy, and a history of allergy), clinical manifestations (mucocutaneous, musculoskeletal, cardiopulmonary, neuropsychiatric, and haematological manifestations, as well as antiphospholipid syndrome and Sjogren’s syndrome), immunological factors [antibodies (Ab) including ANA, anti-dsDNA, anti-Sm, anti-RNP, anti-SSA/Ro, anti-SSB/La and antiphospholipid, and serum complements], and medication administration (glucocorticoid, hydroxychloroquine, aspirin, and low molecular weight heparin) were obtained from medical records.

### Disease activity of SLE and pregnancy outcomes

SLE activity was scored using the SLE-Pregnancy Disease Activity Index (SLEPDAI) [21], and a score > 4 was considered active SLE. The definition of LN met the 1997 ACR criterion [19] and active LN was defined as active urine sediment or proteinuria, where proteinuria was defined by persistent proteinuria > 0.5 g/24 h or random proteinuria ≥3+. Diverse-specific pregnancy outcomes were composited into three types of outcomes, including composite adverse live-birth outcomes [preterm birth, foetal distress, LBW, foetal growth restriction (FGR) and small for gestational age (SGA), birth asphyxiation], composite adverse foetal outcomes (foetal loss and composite adverse live-birth outcomes), and composite adverse maternal outcomes [active SLE, active LN, HDP, gestational diabetes mellitus (GDM), and postpartum haemorrhage (PPH)]. The above outcomes were defined as follows: foetal loss (pregnancy loss including stillbirth, spontaneous miscarriage, and therapeutic/elective abortion), preterm birth (delivery before 37 completed weeks of gestation), foetal distress (a condition during pregnancy or labour in which the foetus shows signs of inadequate oxygenation), LBW (birth weight < 2500 g), FGR (the failure of the foetus to reach its growth potential), SGA (a weight below the 10th percentile for the gestational age), birth asphyxiation (no spontaneous breathing or failure to establish regular breathing within 1 min), HDP (a spectrum of diseases that coexist with pregnancy and hypertension), GDM (a condition in which a woman without diabetes develops high blood sugar levels during pregnancy), and PPH (loss of > 500 ml of blood after a vaginal birth or 1000 ml of blood after a caesarean section within the first 24 h).

### Statistical analysis

Statistical analyses were performed using SPSS (version 24). Measurement data that did not conform to the normal distribution were expressed as median (interquartile range) and compared using the *Mann–Whitney U* test. Enumeration data were presented as the ratio, and hypothesis testing for significant differences was performed using *Pearson’s chi-square* or *Fisher’s exact test*. Logistic regression analysis calculating crude or adjusted *odds ratios* (*ORs*) and their 95% *confidence intervals* (*CIs*) were used to explore the association between cSLE and the risk of active SLE during pregnancy, active LN during pregnancy, and pregnancy outcomes. Variables with *P* <  0.10 in unadjusted analysis were considered in the multivariable logistic regression analysis. When exploring the association of cSLE with both active SLE and active LN during pregnancy, gestational age and in remission for < 6 months before pregnancy were adjusted, while a history of LN was excluded due to potential mediation. For the association between cSLE and gestational outcomes, a history of LN, active SLE during pregnancy, and active LN during pregnancy were excluded due to potential mediation. In model A, factors before pregnancy and demographic profiles were considered, while factors during pregnancy were considered in model B. Statistical significance was set at *P* <  0.05.

## Results

### Comparison of general factors between the cSLE and aSLE groups

Among the 167 pregnancies from women diagnosed with SLE before pregnancy, 22 (13.2%) were cSLE cases (< 18 years of age) and 145 (86.8%) were aSLE cases (≥18 years of age). As shown in Table 1, the gestational age was younger in the cSLE group than that in the aSLE group (23.50 years vs. 29.00 years, *P* <  0.001). The proportion of primiparous patients in the cSLE group was higher than that in the aSLE group (86.4% vs. 61.4%, *P* = 0.022). Meanwhile, the incidence of LN before pregnancy was higher in the cSLE group than in the aSLE group (63.6% vs. 33.1%, *P* = 0.006). However, no significant differences in pre-pregnancy BMI, native place, employment, food or drug allergy history, adverse pregnancy and birth history, caesarean section history, parity after diagnosis of SLE, and SLE in remission for < 6 months before pregnancy were observed between the two groups.

**Table 1:** Comparison of general factors between the cSLE and aSLE groups

Characteristics	cSLE (***N*** = 22)	aSLE (***N*** = 145)	***P*** value
Gestational age, years, median (IQR)	23.50 (20.75, 29.00)	29.00 (26.00, 32.00)	**< 0.001**
SLE duration, years, median (IQR)	10.00 (5.75, 13.50)	5.00 (3.00, 7.00)	**< 0.001**
Pre-pregnancy BMI, kg/m^2^, median (IQR)	19.27 (17.49, 22.09)	20.48 (18.72, 22.04)	0.086
Native place			0.467
Guangdong province	16 (72.7%)	94 (64.8%)	
Others	6 (27.3%)	51 (35.2%)	
Employment			0.758
Unemployed	15 (68.2%)	94 (64.8%)	
Employed	7 (31.8%)	51 (35.2%)	
Parity			**0.022**
Primiparous	19 (86.4%)	89 (61.4%)	
Multiparous	3 (13.6%)	56 (38.6%)	
History of medicine or food allergic	3 (13.6%)	36 (24.8%)	0.248
History of adverse pregnancy and birth	3 (13.6%)	30 (20.7%)	0.626
History of caesarean section	2 (9.1%)	21 (14.5%)	0.725
First pregnancy after diagnosis of SLE	19 (86.4%)	121 (83.4%)	0.972
SLE in remission for < 6 months before pregnancy	1 (4.5%)	14 (9.7%)	0.703
LN before pregnancy	14 (63.6%)	48 (33.1%)	**0.006**

*SLE* systemic lupus erythematosus, *LN* lupus nephritis, *cSLE* childhood-onset systemic lupus erythematosus, *aSLE* adult-onset systemic lupus erythematosus, *BMI* body mass index, *IQR* interquartile range

### Analysis of differences in clinical features and medications between the cSLE and aSLE groups during pregnancy

During pregnancy, the cSLE group had a higher proportion of active SLE (SLEPDAI > 4) than the aSLE group (54.5% vs. 20.7%, *P* = 0.001). Main clinical manifestations during pregnancy in both cSLE and aSLE groups were active LN, haematologic disorders, mucocutaneous disorders, and cardiopulmonary disorders. Active LN (50.0%) was most common in the cSLE group, while haematologic disorders in the aSLE group (33.8%) were the most common. Between the cSLE and aSLE groups, a significant difference in the incidence of active LN was observed (50.0% vs. 17.9%, *P* = 0.001), while the incidences of mucocutaneous disorders, musculoskeletal disorders, cardiopulmonary disorders, liver dysfunction, neuropsychiatric disorders, and haematologic disorder were not significantly different. Pregnant women with cSLE did not have antiphospholipid syndrome or Sjogren’s syndrome, while pregnant women with aSLE had eight cases of antiphospholipid syndrome and seven cases of Sjogren’s syndrome, although no significant difference was identified between the two groups (*P* > 0.05).

Among the immunological indicators, the positive anti-dsDNA Ab rate in the cSLE group was higher than that in the aSLE group, while low complement level and positive Ab levels, including ANA, anti-Sm, anti-RNP, anti-SSA/Ro, anti-SSB/La, and antiphospholipid, were lower in the cSLE group than in the aSLE group. However, among these indicators, only positive anti-dsDNA and anti-SSA/Ro Ab levels were significantly different (*P* <  0.05).

**Fig. 2:**
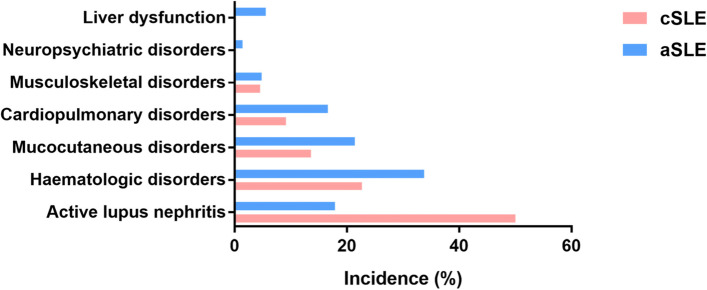
Clinical manifestations of cSLE and aSLE during pregnancy

Regarding medication during pregnancy, the numbers of SLE pregnancies taking glucocorticoids, hydroxychloroquine, low molecular weight heparin and aspirin were 150 (89.8%), 109 (65.3%), 30 (18.0%) and 48 (28.7%) respectively. Between the cSLE and aSLE groups, no significant differences (*P* > 0.05) in the use of glucocorticoids, hydroxychloroquine, low molecular weight heparin, and aspirin were observed. The results are presented in Table 2 and Fig. 2.

**Table 2:** Comparison of the condition and drug treatment between the cSLE and aSLE groups during pregnancy

Condition or drug treatment	cSLE (***N*** = 22)	aSLE ***(N*** = 145)	***P*** value
Active SLE (SLEPDAI > 4)	12 (54.5%)	30 (20.7%)	**0.001**
Active with active LN	11/12 (91.7%)	20/30 (66.7%)	0.202
Mucocutaneous disorders	3 (13.6%)	31 (21.4%)	0.578
Musculoskeletal disorders	1 (4.5%)	7 (4.8%)	1.000
Cardiopulmonary disorders	2 (9.1%)	24 (16.6%)	0.559
Liver dysfunction	0 (0.0%)	8 (5.5%)	0.553
Neuropsychiatric disorders	0 (0.0%)	2 (1.4%)	1.000
Haematologic disorders	5 (22.7%)	49 (33.8%)	0.301
Active LN	11 (50.0%)	26 (17.9%)	**0.001**
Antiphospholipid syndrome	0 (0.0%)	8 (5.5%)	0.553
Sjogren’s syndrome	0 (0.0%)	7 (4.8%)	0.596
Positive ANA Ab	14 (63.6%)	121 (83.4%)	0.056
Positive anti-dsDNA Ab	14 (63.6%)	53 (36.6%)	**0.016**
Positive anti-Sm Ab	0 (0.0%)	25 (17.2%)	0.073
Positive anti-RNP Ab	5 (22.7%)	55 (37.9%)	0.166
Positive anti-SSA/Ro Ab	9 (45.0%)	88 (67.7%)	**0.048**
Missing	2	15	
Positive anti-SSB/La Ab	1 (5.0%)	19 (14.6%)	0.410
Missing	2	15	
Positive antiphospholipid Ab	4 (26.7%)	39 (31.0%)	0.965
Missing	7	19	
Low complement level	14 (63.6%)	81 (56.6%)	0.537
Missing	0	2	
Glucocorticoids	22 (100.0%)	128 (88.3%)	0.188
Dosage > 15	6/22 (27.3%)	27/127 (21.3%)	0.727
Hydroxychloroquine	15 (68.2%)	94 (64.8%)	0.758
LMWH	4 (18.2%)	26 (17.9%)	1.000
Aspirin	5 (22.7%)	43 (29.7%)	0.503

*SLE* systemic lupus erythematosus, *LN* lupus nephritis, *cSLE* childhood-onset systemic lupus erythematosus, *aSLE* adult-onset systemic lupus erythematosus, *SLEPDAI* SLE-Pregnancy Disease Activity Index, *Ab* antibody, *LMWH* low molecular weight heparin

### Association between cSLE and disease activity during pregnancy

As shown in Table 3, cSLE increased the risk of active disease and active LN during pregnancy. Among 167 pregnancies with SLE, 42 (25.1%) had active SLE, and 37 (22.2%) developed active LN during pregnancy. In the unadjusted logistic analysis, the risk of active SLE in pregnant women with cSLE was 4.600 times higher than that in those with aSLE (95%*CI* 1.814–11.664, *P* = 0.001). After adjusting for SLE in remission for < 6 months before pregnancy and gestational age, cSLE increased the risk of active SLE with an *OR* of 4.742 (95%*CI* 1.678–13.405, *P* = 0.003).

**Table 3:** Association between cSLE and disease activity during pregnancy

Disease activity	Univariable analysis		Multivariable analysis	
	***OR*** (95%***CI***)	***P*** value	***OR*** (95%***CI***)	***P*** value
**Active SLE (*N*** **= 42)**				
cSLE	4.600 (1.814–11.664)	**0.001**	4.742 (1.678–13.405)	**0.003**
SLE in remission for < 6 months before pregnancy	7.500 (2.394–23.501)	**0.001**	9.700 (2.954–31.854)	**< 0.001**
Gestational age	0.906 (0.833–0.986)	**0.022**	0.950 (0.867–1.042)	0.276
**Active LN (*N*** **= 37)**				
cSLE	4.577 (1.793–11.685)	**0.001**	4.652 (1.630–13.279)	**0.004**
SLE in remission for < 6 months before pregnancy	4.847 (1.626–14.447)	**0.005**	6.110 (1.952–19.123)	**0.002**
Gestational age	0.911 (0.835–0.994)	**0.037**	0.960 (0.875–1.053)	0.387

*SLE* systemic lupus erythematosus, *LN* lupus nephritis, *cSLE* childhood-onset systemic lupus erythematosus, *OR* odds ratio, *CI* confidence interval

Additionally, the risk of active LN in pregnant women with cSLE was 4.577 times higher than that with aSLE in the unadjusted analysis (95%*CI* 1.793–11.685, *P* = 0.001). After adjusting for SLE in remission for < 6 months before pregnancy and gestational age, cSLE increased the risk of active LN with an *OR* of 4.652 (95%*CI* 1.630–13.279, *P* = 0.004). Moreover, SLE in remission for < 6 months before pregnancy increased the risk of active SLE during pregnancy by 9.700-fold (*P* <  0.001) and the risk of active LN by 6.110-fold (*P* = 0.002).

### Comparison of pregnancy outcomes between the cSLE and aSLE groups

Among the 167 SLE pregnancies, 97 (58.1%) had composite adverse foetal outcomes, and 39 (23.4%) had foetal loss. Among the 142 patients with SLE, excluding therapeutic or selective foetal loss, 14 (9.9%) had foetal loss. Of the 128 SLE pregnancies with live births, there were 14 cases in the cSLE group and 114 cases in the aSLE group. Among the live birth outcomes, 45.3% had composite adverse live-birth outcomes, 22.7% had preterm birth, 2.3% had foetal distress, 7.8% had FGR, 28.9% had LBW, 21.9% had SGA, and 8.6% were asphyxiated. Meanwhile, among the maternal outcomes, 35.9% had composite adverse maternal outcomes, 20.3% had active SLE, 18.0% had active LN, 12.5% had HDP, 9.4% had GDM, and 3.1% had PPH.

Pregnant women in the cSLE group had a higher incidence of composite adverse foetal outcomes, foetal loss, composite adverse live-birth outcomes, foetal distress, SGA, and foetal asphyxiation than those in the aSLE group. Foetal loss without therapeutic or elective abortion, preterm birth, FGR, and LBW indicated the opposite trend. Maternal outcomes in the cSLE group with higher incidence than those in the aSLE group included the following types: composite adverse maternal outcomes, active SLE, activity LN, and PPH. Whereas, HDP and GDM indicated the opposite trend. A significant difference in active SLE and active LN (*P* <  0.05) was identified, while no significant difference in other outcomes was noted (*P* > 0.05), as presented in Table 4.

**Table 4:** Comparison of pregnancy outcomes between the cSLE and aSLE groups

Pregnancy outcomes	cSLE	aSLE	***P*** value
**Foetal outcomes**	***N*** **= 22**	***N*** **= 145**	
Composite adverse foetal outcomes	16 (72.7%)	81 (55.9%)	0.135
Foetal loss	8 (36.4%)	31 (21.4%)	0.122
Foetal loss (without therapeutic/elective abortion)	0/14 (0.0%)	14/128 (10.9%)	0.406
**Live birth outcomes**	***N*** **= 14**	***N*** **= 114**	
Composite adverse live-birth outcomes	8 (57.1%)	50 (43.9%)	0.346
Preterm birth	2 (14.3%)	27 (23.7%)	0.649
Foetal distress	1 (7.1%)	2 (1.8%)	0.296
FGR	1 (7.1%)	9 (7.9%)	1.000
LBW	4 (28.6%)	33 (28.9%)	1.000
SGA	4 (28.6%)	24 (21.1%)	0.764
Asphyxiation	2 (14.3%)	9 (7.9%)	0.764
**Maternal outcomes**	***N*** **= 14**	***N*** **= 114**	
Composite adverse maternal outcomes	8 (57.1%)	38 (33.3%)	0.080
Active SLE	7 (50.0%)	19 (16.7%)	**0.010**
Active LN	7 (50.0%)	16 (14.0%)	**0.003**
HDP	1 (7.1%)	15 (13.2%)	0.830
GDM	0 (0.0%)	12 (10.5%)	0.430
PPH	1 (7.1%)	3 (2.6%)	0.374

*SLE* systemic lupus erythematosus, *LN* lupus nephritis, *cSLE* childhood-onset systemic lupus erythematosus, *aSLE* adult-onset systemic lupus erythematosus, *FGR* foetal growth restriction, *LBW* low birth weight, *SGA* small for gestational age, *HDP* hypertensive disorders in pregnancy, *GDM* gestational diabetes mellitus, *PPH* postpartum haemorrhage

### Association of cSLE with adverse pregnancy outcomes

As described in Table 5, no significant association of cSLE with the risk of composite adverse pregnancy outcomes was identified. The risk of composite adverse foetal outcomes was 2.107 (95%*CI* 0.780–5.692) times higher in the cSLE group than in the aSLE group in the unadjusted analysis. Model 1a adjusted for gestational age, SLE duration, and SLE in remission for < 6 months before pregnancy, and model 1b adjusted for haematologic disorders, low complement level, and aspirin on the basis of model 1a, where cSLE increased the risk of composite adverse foetal outcomes with *ORs* of 2.496 (95%*CI* 0.653–9.542) and 2.285 (95%*CI* 0.549–9.503), respectively.

**Table 5:** Association of cSLE with adverse pregnancy outcomes

Pregnancy outcomes	cSLE		aSLE
	***OR*** (95%***CI***)	***P*** value	
**Composite adverse foetal outcomes (*N*** **= 97)**			
Unadjusted	2.107 (0.780–5.692)	0.142	1 (reference)
Model 1a	2.496 (0.653–9.542)	0.181	1 (reference)
Model 1b	2.285(0.549–9.503)	0.256	1 (reference)
**Composite adverse live-birth outcomes (*N*** **= 58)**			
Unadjusted	1.707 (0.556–5.237)	0.350	1 (reference)
Model 2a	1.348 (0.410–4.434)	0.623	1 (reference)
Model 2b	1.417 (0.419–4.789)	0.575	1 (reference)
**Composite adverse maternal outcomes (*N*** **= 46)**			
Unadjusted	2.667 (0.863–8.237)	0.088	1 (reference)
Model 3a	2.891 (0.917–9.117)	0.070	1 (reference)
Model 3b	3.057 (0.936–9.986)	0.064	1 (reference)

Model 1a: gestational age, SLE duration, and SLE in remission for < 6 months before pregnancy; model 1b: model 1a + haematologic disorders, low complement level, and aspirin

Model 2a: gestational age, and SLE in remission for < 6 months before pregnancy; model 2b: model 2a + haematologic disorders

Model 3a: pre-pregnancy BMI, and SLE in remission for < 6 months before pregnancy; model 3b: model 3a + haematologic disorders

*cSLE* childhood-onset systemic lupus erythematosus, *OR* odds ratio, *CI* confidence interval

For live-birth outcomes, the risk of composite adverse live-birth outcomes was 1.707 (95%*CI* 0.556–5.237) times higher in the cSLE group in the unadjusted analysis. Model 2a adjusted for gestational age and SLE in remission for < 6 months before pregnancy, and model 2b further adjusted for haematologic disorders, in which cSLE increased the risk of composite adverse live-birth outcomes with *ORs* of 1.348 (95%*CI* 0.410–4.434) and 1.417 (95%*CI* 0.419–4.789), respectively.

For maternal outcomes, the risk of composite adverse maternal outcomes was 2.667 (95%*CI* 0.863–8.237) times higher in the cSLE group in the unadjusted analysis. Model 3a adjusted pre-pregnancy BMI and SLE in remission for < 6 months before pregnancy, and model 3b further adjusted for haematologic disorders, where cSLE increased the risk of composite adverse maternal outcomes with *ORs* of 2.891 (95%*CI* 0.917–9.117) and 3.057 (95%*CI* 0.936–9.986), respectively. Nevertheless, none of the above values were significant (*P* > 0.05).

## Discussion

Our study revealed that patients with cSLE during pregnancy had similar characteristics with non-pregnant women with SLE. As expected, the rate of cSLE in women with a history of LN was higher than that of women with aSLE. Here, 13.2% of cSLE pregnancies were identified, in which active SLE (SLEPDAI > 4) and active LN during pregnancy had a high incidence of 54.4% and 50.0%, respectively. Both univariable and multivariable analyses indicated that cSLE was significantly associated with active SLE and active LN during pregnancy. Furthermore, it is known that anti-dsDNA Ab fluctuates with disease activity in patients with SLE and can accumulate in the glomerular and tubular basement membrane by directly binding to self-antigens or indirectly forming immune complexes [22]. Here, a more positive anti-dsDNA Ab was observed in pregnant women with cSLE than in those with aSLE. Thus, cSLE may be more aggressive than aSLE during pregnancy.

No significant association between cSLE and the risk of composite adverse pregnancy outcomes was identified in our study. Patients with cSLE had higher incidences of foetal loss, foetal distress, SGA, asphyxia, and PPH. Foetal loss (without therapeutic/elective abortion), FGR, preterm birth, and LBW, as well as HDP and GDM indicated the opposite trend without significant difference. The population of specific pregnancy outcomes was small, as in many previous studies [23–25] on pregnant women with SLE, due to limited research participants. Hence, the association between cSLE and the risk of adverse pregnancy outcomes was roughly analysed using multivariable analysis. The results indicated that cSLE was not associated with composite adverse foetal outcomes, composite adverse maternal outcomes, or composite adverse live-birth outcomes. In only one published study [26] that addressed a similar issue, 58 (31.18%) cSLE and 128 (68.82%) aSLE pregnancies were included in Mexico. The proportion of cSLE pregnancies was much higher than that in our study, which may explain the differences in ethnic disparities. Although their study has also demonstrated no association of cSLE with risk of adverse pregnancy outcomes, their composition of outcomes was different from our study and lack of general information, such as on pre-pregnancy BMI, maternal history of atopy, and demographic characteristics, may have biased the results. The number of pregnant women with SLE is expected to increase in the future. Using more rigorous protocols and expanding populations with multiple races, further related studies between cSLE and pregnancy outcomes are needed.

LN is the most common manifestation that indicates SLE, and up to 75% of patients with SLE who have flares during pregnancy will have LN [27, 28]. Here, active LN during pregnancy had a high proportion (73.81%) in 42 pregnancies with active SLE. An increasing number of studies have reported that LN and active disease are associated with adverse pregnancy outcomes. A systematic review and meta-analysis [7] of 16 studies, including 1760 pregnancies, indicated that pregnant women with LN had a significant decrease in live births (*OR* = 0.62), while a significant increase in preterm births (*OR* = 1.92) and FGR (*OR* = 1.43). LN history (*RR* = 1.62), active SLE in pregnancy (*RR* = 2.98), and active LN in pregnancy (*RR* = 1.78) significantly increased the risk of preterm birth, as shown in another meta-analysis of 24 observational studies [29]. Furthermore, cSLE was related to a history of LN, active SLE in pregnancy, and active LN in pregnancy, so cSLE may also have indirect effects on the risk of adverse pregnancy outcomes through LN and active disease. Based on the above, further studies focused on such patients and their management are required in the future.

The molecular pathogenesis of the difference between cSLE and aSLE remains unclear. Omarjee et al. [30] have found an association between cSLE and single-gene mutations. Webber et al. [31] have reported that SLE risk loci played an important role in LN risk in patients with cSLE compared with those with aSLE. These findings highlight the importance of genetic aetiology in patients with cSLE. Genetic factors might also function in pregnancy; however, there are no studies on the molecular evolution of cSLE patients during pregnancy. It is known that the human placenta is the most important foetal development organ during pregnancy, which mediates nutrient and waste exchange between the mother and the embryo/foetus by preventing its rejection by the maternal immune system [32]. In recent decades, the role of the placenta in the risk of adverse pregnancy outcomes like FGR and preeclampsia in general pregnancies has been controversial [33–35]. Interestingly, The expression of some molecules in the placenta of SLE patients has been reported to be higher than that in control cases, including complement split product C4d, activated low-density granulocytes, and myeloperoxidase [36–38]. Hence, placenta-related studies may be the direction of future research to further explore the association between cSLE and pregnancy outcomes.

The current study has some limitations. First, it is a single-centre study composed of Han Chinese women, which ensures data homogeneity but could be a limitation for extension to other population groups. Second, the limitation of size of the sample, especially of patients with cSLE, may not allow a robust statistical analysis of the factors potentially associated with an adverse maternal-foetal outcome. Third, the information in our study was retrospectively obtained from medical records in hospitals, and primary data entry into the medical records was not standardised. Accordingly, the accuracy and truthfulness of some data could not be verified. For example, data in our study suggested that none of the women drank alcohol or smoked cigarettes, which may not be true. This is because the template with no drinking and no smoking will be retained in medical records if clinicians do not ask. Forth, although the baseline information of pregnant women collected in our study was more than that of many other studies [23–26], socioeconomic status, lifestyle, exercise, and dietary habits were not available, which should be considered in future studies. These indicators could influence the association between cSLE and outcomes during pregnancy. Moreover, a diagnostic bias may exist. For instance, the differential diagnosis between active LN and PE during pregnancy remains difficult owing to similar signs and laboratory tests. It has been recently demonstrated that evaluation of serum VEGF, PlGF, and sFlt-1 levels can differentiate between preeclampsia, inactive SLE, and active LN during pregnancy [39]. Using such auxiliary diagnostic indicators can further improve accuracy and convincing results in SLE-related studies during pregnancy. As the number of pregnant women with SLE is expected to increase in the future, therefore, rigorously designed prospective multi-centre studies are required.

## Conclusion

Patients with cSLE during pregnancy had similar characteristics to non-pregnant women with SLE, where cSLE was more aggressive than aSLE. Although no significant association between cSLE and the risk of composite adverse foetal/maternal outcomes was observed, cSLE may have indirect effects on the risk of adverse pregnancy outcomes through LN and active disease. Thus, a focus on such patients during pregnancy is still needed. Rigorously designed prospective multi-centre studies on pregnant patients with cSLE are required to provide guidance for the management of pregnant women with SLE and improve their pregnancy outcomes.

## Data Availability

Data and materials were obtained from medical records in hospitals.

## Abbreviations

SLE: Systemic lupus erythematosus
LN: Lupus nephritis
aSLE: Adult-onset systemic lupus erythematosus
cSLE: Childhood-onset systemic lupus erythematosus
ACR: American College of Rheumatology
SLEPDAI: SLE-Pregnancy Disease Activity Index
FGR: Foetal growth restriction
LBW: Low birth weight
SGA: Small for gestational age
HDP: Hypertensive disorders in pregnancy
GDM: Gestational diabetes mellitus
PPH: Postpartum haemorrhage
Ab: Antibody
BMI: Body mass index
